# FROM TUSSIS TO TESTICULAR PAIN, A CASE REPORT ON ACUTE IDIOPATHIC INTRATESTICULAR HEMORRHAGE

**DOI:** 10.51894/001c.122906

**Published:** 2024-08-30

**Authors:** Alexander Haberman

**Affiliations:** 1 Urology Surgery Residency Sparrow Hospital

## 41

### INTRODUCTION

Intratesticular hemorrhage (ITH) is a rare etiology typically associated with blunt trauma, but can be associated with malignancy. The most common malignancy in men aged 20-40 are germ cell testicular tumors, care must be taken to rule out malignancy in any scrotal complaint. Multiple case reports demonstrating autoimmune, anticoagulant use, and benign testicular lesions as an etiology have also been described as sources for ITH. Few case reports describe a spontaneous idiopathic mechanism. Treatment can range from a conservative approach with serial imaging to surgical intervention requiring scrotal exploration with possible removal of the testicle, which may impact future fertility and testosterone levels.

### CASE DESCRIPTION

A pubmed search was conducted for ITH etiologies and management. Acute ITH is scarcely reported in the literature and there has been none reported with any scrotal imaging prior to the event. Furthermore, no case reports exist demonstrating testicle sparing surgical techniques for ITH. A 34 year old male with no prior medical history and no medication use presented to the emergency department due to achy right testicle pain. Due to resolution of pain with a negative scrotal ultrasound, he was discharged home. The following day, the patient coughed and suddenly developed severe right testicle pain, refractory to opioids and ketamine. Repeat US demonstrated a 2.9 x 2.6 cm avascular lesion within the right testicle. Basic labs were unremarkable. He was taken emergently for a testicular exploration via an inguinal approach, where 3cm of clot was removed from within the tunica albuginea and an arterial bleed was controlled. Debridement of nearby tissue demonstrated hemorrhagic testicular tissue with degenerative and early necrosis, no signs of malignancy. The tunica was able to be closed primarily and testicle delivered back into the scrotum. He was discharged several hours later.

### DISCUSSION

Idiopathic ITH has been scarcely reported in the literature; there has been no case reports with any imaging prior to the event, nor any with testicular sparing surgical techniques. Despite negative pathology, this case serves as a reminder to always rule out malignancy as an etiology for ITH and if operative intervention is taken, to plan accordingly.

